# Whether, or under what conditions? A configurational approach to AI-supported antimicrobial stewardship in sub-Saharan Africa

**DOI:** 10.3389/frabi.2026.1954916

**Published:** 2026-10-12

**Authors:** Abdulrasheed Ibrahim

**Affiliations:** Department of Surgery Ahmadu Bello University (ABU) and Ahmadu Bello University Teaching Hospital (ABUTH), Zaria, Kaduna State, Nigeria

**Keywords:** antimicrobial stewardship, artificial intelligence, configurational analysis, qualitative comparative analysis, sub-Saharan Africa

## Introduction

Sub-Saharan Africa carries a disproportionate share of the global burden of antimicrobial resistance. Western sub-Saharan Africa has the highest death rate attributable to resistance of any world region, at 27.3 per 100,000 (Antimicrobial Resistance Collaborators, 2022). Yet the region has some of the thinnest data and diagnostic infrastructure on which modern stewardship depends (Kapatsa et al., 2025). This is the setting into which artificial intelligence is now being introduced. Machine learning tools are being developed to predict resistance, guide empirical therapy, and flag inappropriate prescriptions, promising data-driven stewardship in exactly the settings where the need is greatest (Matsuo et al., 2026).

The prevailing question about these tools is whether they improve stewardship. That question asks for an average effect. Averaged across hospitals with varying infrastructure, artificial intelligence’s effect on stewardship may look small, and be misread as evidence that it is not ready for African hospitals. More likely, its effectiveness depends on data and infrastructure that some hospitals have and others do not, so judging it by an average becomes a question of equity.

This calls for a different question. The success of AI-supported stewardship in sub-Saharan Africa will not be settled at the level of the technology; it will turn on particular combinations of enabling conditions. It should therefore be studied configurationally, asking under what combinations of infrastructural, epidemiological, and human conditions it works, rather than evaluating it for an average effect.

## AI-supported stewardship is a bundle of preconditions

An artificial intelligence tool is not a single intervention, but a bundle of preconditions that must hold together for the tool to help. These preconditions span three dimensions, and each is unevenly met across sub-Saharan Africa.

The first is infrastructural. A predictive tool needs digital records, reliable connectivity, and local microbiology data to train on and to act upon. A recent systematic review of stewardship in sub-Saharan Africa found limited diagnostic and laboratory infrastructure, weak data systems, and underfunded services to be amongst the most common barriers (Kapatsa et al., 2025). Where these are absent, a tool has nothing to read.

The second is epidemiological. A model trained on one population may not transfer to another. That this is not hypothetical is shown by a hepatitis B tool developed on Nigerian data, which performed well in a Nigerian cohort but far less well in an Australian one (Ajuwon et al., 2023). Pathogen mixes, resistance patterns, and comorbidities differ between settings, so local validity is itself a precondition, not a given.

The third is human. An accurate model changes nothing if it is not trusted, not integrated into the clinical workflow, or not supported by training and governance. Kapatsa and colleagues found insufficient staff training, weak governance, and limited leadership to be recurring obstacles (Kapatsa et al., 2025). In many settings these human conditions, not the technology, decide whether a tool is used at all.

These preconditions co-occur unevenly, both between countries and within them. A tertiary centre in a capital city may meet most of them, whilst a district hospital in the same country meets few. No single average adequately captures this variation.

## Why the average-effect question misjudges sub-Saharan Africa

Net-effects evaluation estimates an average effect across cases, assuming the intervention acts more or less independently of its context. For an infrastructure-dependent intervention, that assumption fails, because the conditions are interdependent. An accurate model achieves little without clinician trust, trust is untested without data to run the model, and data remains unused without a workflow that acts on it (Movsisyan et al., 2019). What matters is not the average, but which combinations of conditions are necessary or sufficient for the tool to help.

For sub-Saharan Africa, this is not only a methodological point. An average taken across dissimilar settings does not merely lose information; it can systematically disadvantage the region. A pooled result that looks modest would be reported as a verdict on the technology, when it reflects the uneven presence of the conditions the technology needs. Average-effect framing quietly converts an infrastructure gap into a judgement about whether artificial intelligence suits African hospitals. A configurational framing prevents that error, by asking where, and under what conditions, the tool actually works.

## The configurational approach in practice

Configurational analysis, and Qualitative Comparative Analysis in particular, is a method suited to interventions of this kind (Ragin, 2008). It treats each deployment as a case, scored on a shared outcome and on a set of conditions. The outcome would ideally be a reduction in inappropriate prescribing or in resistance, the shared goal. The conditions would span the three dimensions: infrastructural (data-system maturity and laboratory capacity), epidemiological (local model validation), and human (clinician trust and training, workflow integration, and governance).

The method then asks configurational questions. Is local microbiology data present in every successful deployment, making it necessary? Which combinations of conditions are together sufficient for success? Configurational methods have already been applied across public health interventions (Hanckel et al., 2021). In an integrated mental healthcare trial in South Africa, this approach identified which combinations of conditions produced successful implementation (Janse van Rensburg et al., 2021). A cluster-randomised physical activity programme was analysed the same way, to separate the pathways that led to success from those that did not (Kien et al., 2018).

Configurational analysis also allows for equifinality, meaning that more than one combination of conditions can produce the same outcome (Ragin, 2008). A hospital with limited records might succeed with a simpler, well-validated AI tool, a functioning laboratory, and strong clinician trust. Another, with richer data and connectivity, might succeed with a more data-intensive system. Both reach the goal through AI, by different routes. Equifinality therefore reframes the problem. The question is not whether a hospital can replicate one ideal, data-rich deployment, but which combination of conditions would let AI-supported stewardship work there.

For a hospital like mine, that shift matters. It replaces a single, data-rich template with guidance about what can be done now, at the level of infrastructure actually available. It turns the statement that a hospital lacks the infrastructure for artificial intelligence into a question about which combination of conditions would let it begin.

## Discussion

Several practical challenges remain. Few AI-supported stewardship programmes in the region have been studied configurationally, so the evidence base is thin. Stewardship outcomes are reported inconsistently, which makes studies difficult to compare (Yousuf et al., 2023). Each enabling condition must also be scored by clear, stated rules. Laboratory capacity, for example, might be scored as full where a functioning microbiology service is always available, partial where testing is intermittent, and absent where none exists. Clinician trust might be scored by whether prescribers act on a tool’s advice routinely, occasionally, or not at all. Reporting these rules openly lets others see how each hospital was judged. It also lets the analysis show what a hospital needs, whether more laboratory capacity or greater clinician confidence, before an AI tool can help there. These are real challenges, but they argue for better reporting, not for abandoning the question.

The remedy is within reach. Studies of AI-supported stewardship in sub-Saharan Africa should record not only whether a tool worked, but which conditions were present, and which combinations accompanied success. Such reporting would make configurational analysis possible as the evidence grows, and it builds naturally on implementation research already under way in the region (Kapatsa et al., 2025).

The question for AI-supported stewardship in sub-Saharan Africa is not whether it works, but under what conditions. Answering that question configurationally is at once the sounder science and the fairer one, because it distinguishes a technology that has failed from a hospital that was never resourced to run it. That distinction is what turns a global template into local, achievable guidance.

## References

[B4] Ajuwon B. I. Richardson A. Roper K. Lidbury B. A . (2023). Clinical validity of a machine learning decision support system for early detection of hepatitis B virus: a binational external validation study. Viruses 15, 1735. doi: 10.3390/v15081735 37632077 PMC10458613

[B1] Antimicrobial Resistance Collaborators (2022). Global burden of bacterial antimicrobial resistance in 2019: a systematic analysis. Lancet 399, 629–655. doi: 10.2139/ssrn.6712818 37500506

[B7] Hanckel B. Petticrew M. Thomas J. Green J. (2021). The use of Qualitative Comparative Analysis (QCA) to address causality in complex systems: a systematic review of research on public health interventions. BMC Public Health 21, 877. doi: 10.1186/s12889-021-10926-2 33962595 PMC8103124

[B8] Janse van Rensburg A. Kathree T. Breuer E. Selohilwe O. Mntambo N. Petrus R. . (2021). Fuzzy-set qualitative comparative analysis of implementation outcomes in an integrated mental healthcare trial in South Africa. Glob. Health Action 14, 1940761. doi: 10.1080/16549716.2021.1940761 34402770 PMC8381905

[B2] Kapatsa T. Bwanali A. N. Kambewa L. N. Mkandawire V. Mwale G. Harawa G. . (2025). Assessing the implementation determinants of antimicrobial stewardship programmes in sub-Saharan Africa through the complexity lens. A CFIR-guided systematic review. Front. Microbiol. 16, 1660778. doi: 10.3389/fmicb.2025.1660778 41347245 PMC12672479

[B9] Kien C. Grillich L. Nussbaumer-Streit B. Schoberberger R. (2018). Pathways leading to success and non-success: a process evaluation of a cluster randomized physical activity health promotion program applying fuzzy-set qualitative comparative analysis. BMC Public Health 18, 1386. doi: 10.1186/s12889-018-6284-x 30563481 PMC6299632

[B3] Matsuo T. Nigo M. Borgonovo F. Yoshida H. Tande A. J. Berbari E. F. . (2026). Artificial intelligence in antimicrobial stewardship: prediction, clinical applications, and implementation challenges. Clin. Microbiol. Infect. doi: 10.1016/j.cmi.2026.07.032 42521015

[B5] Movsisyan A. Arnold L. Evans R. Hallingberg B. Moore G. O'Cathain A. . (2019). Adapting evidence-informed complex population health interventions for new contexts: a systematic review of guidance. Implement Sci. 14, 105. doi: 10.1186/s13012-019-0956-5 31847920 PMC6918624

[B6] Ragin C. C. (2008). Redesigning Social Inquiry: Fuzzy Sets and Beyond (Chicago: University of Chicago Press).

[B10] Yousuf S. Rzewuska M. Duncan E. Ramsay C. (2023). Identification of outcomes reported for hospital antimicrobial stewardship interventions using a systematic review of reviews. JAC-Antimicrob. Resist. 5, dlac127. doi: 10.1093/jacamr/dlac127 36601549 PMC9806591

